# Operationalizing Patient and Public Involvement in a Multicenter Study on Hospital Nutrition and Dysphagia in Older Adults: The NUTRIFAG Case Study

**DOI:** 10.3390/healthcare14203486

**Published:** 2026-10-16

**Authors:** Arturo Garcia-Garcia, Cristina Carretero-Randez, Rafaela Camacho-Bejarano, María Teresa Roldán-Chicano, María Trinidad Castillo-García, Joan Blanco-Blanco, María Isabel Orts-Cortés, the NUTRIFAG Group

**Affiliations:** 1 PhD Programme in Health Sciences, Nursing and Community Health Team, Department of Nursing, University of Alicante, Ctra. de San Vicente s/n, 03690 San Vicente del Raspeig, Spain; agg380@alu.ua.es (A.G.-G.); trinidad.castillo@ua.es (M.T.C.-G.); 2 Department of Nursing, Faculty of Health Sciences, Universidad Europea de Valencia, Paseo de la Alameda 7, 46010 Valencia, Spain; 3 Hospital Emergency Department, Hospital Universitario del Vinalopó, Carrer Tonico Sansano Mora 14, 03202 Elche, Spain; 4 Burns Unit, University and Polytechnic Hospital La Fe, Avenida Fernando Abril Martorell 106, 46026 Valencia, Spain; carreterorandez.cristina@gmail.com; 5 Nursing Research Group: Infection, Inflammation and Chronicity (IIS La Fe), Hospital Universitario y Politécnico La Fe, Avenida Fernando Abril Martorell 106, 46026 Valencia, Spain; 6 Nursing and Healthcare Research Unit (Investén-Isciii), Instituto de Salud Carlos III, Av. de Monforte de Lemos 5, 28029 Madrid, Spain; isabel.orts@ua.es; 7 Department of Nursing, Faculty of Nursing, University of Huelva, Campus El Carmen, Avenida 3 de Marzo s/n, 21071 Huelva, Spain; 8 Research Network on Chronicity, Primary Care and Health Promotion (RICAPPS), Gran Via Corts Catalanes 587, 08007 Barcelona, Spain; 9 Department of Nursing, University of Murcia, Campus de Ciencias de la Salud, Av. de la Real Academia de Medicina 4, 30203 Cartagena, Spain; mteresa.roldan@um.es; 10 Biomedical Research Institute of Murcia Pascual Parrilla (IMIB), Pabellón Docente del Hospital Clínico Universitario Virgen de la Arrixaca, C/Campo 12, 30120 El Palmar, Spain; 11 Department of Nursing, Institute for Health and Biomedical Research (ISABIAL, Group 23), University of Alicante (BALMIS), Ctra. de San Vicente s/n, 03690 San Vicente del Raspeig, Spain; 12 Dr. Balmis University General Hospital, Calle Pintor Baeza 12, 03010 Alicante, Spain; 13 Research Group on Society, Health, Education and Culture of Care (GESEC), Faculty of Nursing and Physiotherapy, Universitat de Lleida, 25198 Lleida, Spain; joan.blanco@udl.cat; 14 Research Group of Health Care (GReCS), Biomedical Research Institute of Lleida-Dr. Pifarré Foundation, IRBLleida, 25198 Lleida, Spain; 15 CIBER of Frailty and Healthy Ageing (CIBERFES), Instituto de Salud Carlos III, Av. Monforte de Lemos 3-5, 28029 Madrid, Spain

**Keywords:** patient participation, community participation, deglutition disorders, nutritional status, aged

## Abstract

**Highlights:**

**What are the main findings?**

- A 17-member Citizen Advisory Group bringing together patients, family caregivers, patient associations, clinicians, hospital managers, and catering leads was successfully embedded in a multicenter hospital study on nutrition and dysphagia in older adults.
- Patient and public involvement produced documented refinements to dissemination materials, including an easy-to-read layer, clearer posture cues, QR codes linking to information sources, and more accessible videos.

**What are the implications of the main findings?**

- Structured, low-cost CAG model is feasible in complex multicenter inpatient settings and produced documented refinements intended to make health information clearer and more accessible for older patients and caregivers.
- Early stakeholder engagement, clear role definition, and concise onboarding are key enablers that are transferable to other hospital services managing nutrition and dysphagia.

**Abstract:**

**Background/Objectives:** Malnutrition and dysphagia are common and interrelated conditions among hospitalized older adults and are associated with poorer clinical outcomes. Patient and public involvement (PPI) has been increasingly recommended to improve the relevance, usability, and implementation of healthcare research and interventions; however, structured approaches to integrating PPI in hospital research on nutrition and dysphagia remain limited. This study aimed to describe how PPI was designed and embedded within the multicenter NUTRIFAG project and to identify practical lessons for its implementation. **Methods:** A community case study was conducted within NUTRIFAG, a prospective observational multicenter project assessing nutritional status and dysphagia risk among adults aged ≥65 years admitted to medical and surgical wards in nine Spanish public hospitals. A Citizen Advisory Group (CAG) was embedded in NUTRIFAG as a structured PPI component, and the documentation generated during the PPI process was compiled and synthesized using the GRIPP2 Long Form (GRIPP2-LF) reporting framework. **Results:** The CAG comprised 17 stakeholders representing patients and family caregivers, patient associations, healthcare professionals, hospital management, and hospital food and catering services. Engagement occurred mainly during the design and dissemination phases and included three virtual meetings supported by iterative email exchanges. PPI contributed to several refinements in dissemination materials, including clearer messaging, the addition of a reading-easy layer, improved posture cues, clearer separation of signs and symptoms, incorporation of QR codes linking to information sources, and improved video accessibility through captions and full-screen display. **Conclusions:** Embedding a diverse stakeholder advisory group within a multicenter hospital study was feasible and generated documented refinements to communication materials and dissemination processes. Structured PPI may contribute to clearer and potentially more usable healthcare resources for older patients in hospital settings.

## 1. Introduction

Population aging has accelerated across Europe and Spain, intensifying the clinical and organizational pressures on health systems. According to national demographic indicators, Spain’s aging index [1] has increased steadily over recent decades, reaching 148.05 in 2025, reflecting a sustained expansion of the older population relative to younger cohorts. This demographic shift coincides with epidemiological patterns showing that, among adults aged ≥50 years in 15 European countries, the prevalence of multimorbidity (≥2 chronic diseases) rose consistently between 2004 and 2017, with particularly pronounced increases in Central Europe and Spain [2]. Currently, more than 50 million Europeans live with two or more chronic conditions, an upward trend driven by population aging and improved survival [3]. In Spain, this burden has also grown over time, with evidence of rising multimorbidity in older adults despite organizational improvements that have mitigated, but not reduced, its overall impact [4].

Malnutrition is highly prevalent among hospitalized older adults and represents a major determinant of clinical vulnerability. In individuals aged ≥70 years, approximately one-third are admitted in a well-nourished state, while 18% present with malnutrition and 45% are at risk at hospital admission [5]. Evidence from studies in hospitalized older populations documents similarly elevated levels, with 35–65% of patients classified as malnourished or at nutritional risk [6,7]. These proportions illustrate a substantial and persistent burden that contributes to adverse clinical trajectories, including increased complications, prolonged length of stay, and poorer overall outcomes.

Dysphagia is a critical, yet often underrecognized, contributor to clinical decline in hospitalized older adults. Evidence from reviews of hospitalized older populations identifies dysphagia as one of the most important predictors of malnutrition, with both conditions exerting mutually reinforcing effects that accelerate functional deterioration [8]. In frail hospitalized older adults, malnutrition has also been identified as a key risk factor for the development of dysphagia, establishing a bidirectional cycle that compounds vulnerability and exacerbates adverse outcomes [9].

Accurate and actionable nutritional assessment in hospitalized older adults requires clinical tools that are both feasible and standardized. Reviews of methods used in acute and critical-care settings highlight substantial variability in how dietary intake is recorded, emphasizing that greater validation and standardization are needed for intake monitoring to serve as a reliable basis for clinical decision-making [10]. In geriatric populations, the combined use of structured screening instruments such as the Eating Assessment Tool-10 (EAT-10), a questionnaire that screens swallowing difficulty, and standardized dietary frameworks such as the International Dysphagia Diet Standardisation Initiative (IDDSI), which classifies food and fluid consistency in dysphagia care, has been shown to enhance patient safety, improve oral intake, and support better nutritional status [11].

PPI strengthens the scientific and practical value of healthcare research and interventions by aligning priorities, materials, and procedures with the needs of intended users. Systematic reviews show that PPI contributes to formulating research questions that better reflect user priorities, producing more comprehensible information materials, selecting data-collection methods that are more acceptable, and developing recruitment strategies that are more feasible and context-appropriate [12]. Evidence also associates PPI with improved implementation, sustainability, and applicability of programs in community and long-term care settings, where participatory approaches enhance cultural relevance, trust, and the effectiveness of dissemination pathways such as co-authorship, presentations, and community networks [13]. Despite these benefits, major gaps remain, including limited formal evaluation, inconsistent reporting, and the risk of superficial or purely consultative participation [14].

The expansion of PPI across health research has been accompanied by highly variable and incomplete reporting, limiting the ability to determine what works, for whom, and under which conditions [15]. The need to address these inconsistencies led to the development of the original Guidance for Reporting Involvement of Patients and the Public (GRIPP) checklist in 2011, designed to improve the quality, transparency, and consistency of PPI reporting [16]. Building on this initial effort, Guidance for Reporting Involvement of Patients and the Public version 2 (GRIPP2) was created in 2017 through systematic reviews and an international Delphi process to establish the first evidence-based, consensus-driven international guidance specifically aimed at strengthening the rigor and comparability of the PPI evidence base [15]. Subsequent reviews in areas such as patient safety, oncology, and research conducted in low- and middle-income settings have used GRIPP2 to assess the frequency and quality of PPI reporting, emphasizing that more systematic application of these standards would enable the development of a richer and more cumulative body of evidence [17].

In the domain of hospital malnutrition and dysphagia in older adults, the integration of PPI remains insufficiently systematized. Recent protocol-level work highlights the absence of clear guidance on how to meaningfully engage malnourished or dysphagic older patients as co-researchers, despite the recognized need to incorporate their experiential knowledge into study design and implementation [18,19].

Accordingly, the present study aimed to describe how PPI was designed and embedded within the multicenter NUTRIFAG project, to appraise its role across the different phases of the study, and to distill practical lessons to inform scalable, meaningful PPI in hospital nutrition and dysphagia care for older adults.

## 2. Materials and Methods

### 2.1. Study Design

We conducted a community case study of a PPI structure embedded within NUTRIFAG, a prospective, observational, multicenter project in Spain assessing nutritional status and dysphagia risk among adults aged ≥65 years admitted to medical and surgical wards across public hospitals.

### 2.2. Target Population and Study Sites

The case study was anchored in the host project’s inpatient population: adults aged ≥65 years admitted to medical and surgical wards in Spanish public hospitals, which provided the clinical and organizational context within which the PPI activities were embedded.

The host project, NUTRIFAG, is a prospective, observational, multicenter study evaluating nutritional status and dysphagia risk in this population. It was coordinated by the Alicante Institute for Health and Biomedical Research (ISABIAL) and funded through competitive public funding from the Instituto de Salud Carlos III (grant PI21/00738), with co-funding from the European Union. The host protocol received approval from the relevant Research Ethics Committees prior to the initiation of any procedures.

The study operated across nine hospitals situated in seven autonomous communities (Andalusia, Aragón, Catalonia, Valencian Community, Canary Islands, Murcia, and Navarre), ensuring representation of diverse hospital settings within Spain’s public health system. For temporal context, the host study spanned 2022–2024, during which the participating hospitals contributed eligible inpatient episodes and supported associated PPI engagement activities.

These details are reported here solely to situate the PPI case within its real-world care environment and to provide the organizational and ethical context necessary for transparency and reproducibility.

### 2.3. Patient and Public Involvement

The CAG was conceived as a structured PPI component embedded in the NUTRIFAG study from its design phase. Its members were not research subjects, but public contributors who brought experiential, professional, organizational and service-delivery knowledge to inform selected aspects of the study design, communication materials and dissemination strategy. In line with NIHR guidance [20], which encourages involving more than one public contributor and offering different levels of involvement across the research lifecycle, the group was constituted to allow contributors to select the intensity and stage of engagement most appropriate to their circumstances.

Members were recruited purposively. The research team first defined the stakeholder profiles needed to inform the study. Eligible contributors were subsequently identified and invited with the support of the local working groups at each hospital participating in the NUTRIFAG project, based on their availability and relevance to the predefined profiles. The CAG was established as a standing advisory structure bringing together individuals with diverse lived, professional and organizational perspectives. Consistent with the literature on community and stakeholder advisory boards in participatory health research [21], we use the term CAG to reflect this mixed composition. For clarity, throughout the manuscript the term ‘public contributors’ refers to patients, family caregivers and patient-association representatives, ‘stakeholders’ to the group as a whole, and ‘PPI’ to the overall approach.

Participation levels were defined according to the type and depth of involvement, distinguishing four degrees: information, consultation, collaboration, and partnership/alliance [22].

- Information consisted of obtaining general input, opinions, or experiences related to specific tasks or topics, such as identifying or validating the study topic through surveys or informational meetings.
- Consultation entailed the collection of feedback and advice on concrete research questions or activities, for example, reviewing study documents, assessing the relevance of content, or appraising dissemination materials.
- Collaboration involved working directly with PPI participants throughout the study or at specific stages to ensure that their expectations and concerns were adequately reflected in project development.
- Finally, partnership/alliance established a shared-leadership relationship between public representatives and the research team, with joint decision-making on the research process, for example, as members of a steering or oversight committee.

Roles assumed by members of the CAG encompassed advisor/subject-matter expert, personal participant, and co-investigator [22]. The advisor/expert role implied offering guidance and direction based on individual or collective experience, representing diverse viewpoints such as professional associations or advisory committees. Personal participation referred to contributions from individuals not directly affected by the study topic who provided perspectives and comments grounded in their experience or knowledge of the healthcare context. As co-investigators, PPI members were considered equal partners whose expertise contributed substantively to study development and to the interpretation of findings.

Stages of the research process in which the CAG could be integrated included: needs identification and prioritization of research topics; study design; development or review of study documents; methods definition; recruitment strategies; data collection and analysis; validation of results; and scholarly dissemination (including potential co-authorship). Collaboration methods comprised co-design of interventions, ongoing facilitation and feedback, participation in learning health collaboratives, peer-led interventions, and co-led dissemination activities alongside the research team [22].

### 2.4. Materials

We used the GRIPP2 [15] as the primary instrument to capture and structure all PPI information in this case study. GRIPP2 is an evidence-based, consensus guideline developed through systematic reviews and an international Delphi process to improve the quality, transparency, and comparability of PPI reporting. We adopted the Long Form (GRIPP2-LF) as our main data-collection template.

GRIPP2 is available in two complementary formats. The LF provides a comprehensive item set (e.g., aims, context, methods, stages and nature of involvement, capture/measurement of impact, outcomes, resource/economic considerations, and reflections/critical perspective) intended for studies where PPI is a primary focus. The Short Form (GRIPP2-SF) condenses reporting to five items—aim, methods, results/outcomes, overall influence, and critical perspective—and is recommended when PPI is secondary to another primary objective [15]. For this manuscript, which centers on the design, governance, and impact of a CAG within a hospital context, we selected GRIPP2-LF to ensure granularity, traceability, and replicability of PPI decisions and effects.

### 2.5. Data Analysis

Data analysis consisted of a structured thematic synthesis of the contributions generated during the PPI process, guided by the GRIPP2-LF framework. The research team compiled the materials generated, including meeting notes, written comments, questionnaire responses, email exchanges and successive versions of dissemination materials. These documents were reviewed to identify CAG contributions, suggested changes, implemented modifications and reflections on the involvement process.

The project documents produced within NUTRIFAG (study materials and dissemination resources) were reviewed by each member of the CAG, whereas the synthesis of the contributions they generated was carried out by the research team. Following the principles of thematic analysis [23], contributions were read repeatedly, coded, and grouped into themes; similar suggestions were merged and duplicates removed. Coding and grouping were performed collaboratively and discussed by the research team until consensus was reached, and any discrepancies were resolved through joint discussion. To support reflexivity and an audit trail, each synthesized item was linked to its source document and to the corresponding tracked-change draft, preserving traceability between the original stakeholder contributions and the final categories.

Relevant information was then mapped deductively to the corresponding GRIPP2-LF items. Free-text contributions were summarized descriptively by grouping similar suggestions, removing duplications and linking each synthesized finding to its documentary source. Structured questionnaire responses, when available, were summarized narratively and integrated into the same reporting matrix.

This approach was chosen because the aim of the case study was not to generate new qualitative theory, but to document how PPI was implemented, what it contributed to the study process and which lessons could inform future research.

The completed GRIPP2-LF reporting checklist, which maps each item to the section of the manuscript in which it is reported, is provided in Table 1 as a transparency and reproducibility aid rather than as a presentation of study findings.

## 3. Results

### 3.1. Group Composition

The CAG comprised 17 members representing five stakeholder groups: patients and family caregivers (*n* = 3), patient-association representatives (*n* = 3), healthcare professionals (*n* = 5, covering speech-language pathology/phoniatrics, physiotherapy, nursing care assistance, social work, and dietetics-nutrition), health management and administration (*n* = 3, covering care management, economic management, and coordination), and hospital food and catering leads (*n* = 3). This composition was deliberately designed to bring together experiential, professional, organizational, and service-delivery knowledge, ensuring that the group could contribute multiple perspectives to the study.

Although contributors with direct patient or caregiver experience (*n* = 3) were outnumbered by professional and organizational stakeholders, facilitation was designed to prevent their perspective from being diluted: the facilitator guaranteed a speaking turn to each stakeholder group in every session, and patients and family caregivers were given the opportunity to provide the final input in each discussion round, so that their criterion served as the core reference for the group’s conclusions. The CAG is therefore best understood as a mixed, multistakeholder advisory structure in which patient and caregiver voices were deliberately protected, rather than as a group composed solely of public contributors [21]. Convening such a diverse, multistakeholder group was a deliberate and innovative feature of the design, in line with the literature on community and stakeholder advisory boards [21], intended to capture a broader range of perspectives than a single-stakeholder panel while safeguarding the patient and caregiver voice.

### 3.2. Stages and Participatory Mechanisms

PPI occurred at two principal stages—design and dissemination—and spanned a continuum of involvement, including information (briefings), consultation (document/usability review), collaboration (iterative co-redesign), without reaching the level of partnership or shared leadership as defined in Section 2.3.

Engagement was structured around three virtual meetings of approximately 60 min each, within which semi-structured interview prompts and focus-group facilitation were used to elicit feedback systematically. Between sessions, iterative email exchanges supported the drafting and approval of materials, ensuring continuity and traceability of decisions across the process. In addition to the virtual meetings, an in-person meeting was held after the first virtual session, once contributors had been identified and had agreed to participate, to consolidate the group and clarify roles, and to qualitatively explore the contributors’ views on and experience of the participatory process.

### 3.3. Operation of the Advisory Group

Operationally, the CAG functioned as a standing advisory structure coordinated by the investigative team, with minutes and tracked-change drafts documenting decisions and revisions. Participation was voluntary; contributors received no payment or reimbursement, and the predominantly virtual format minimized out-of-pocket costs. No formal post-implementation re-test was undertaken; the contributions and resulting modifications were documented and mapped onto GRIPP2-LF items, which served as a reporting framework.

The contributors’ own experience of the participatory process was also gathered qualitatively at the in-person meeting. Reflective learning emphasized what worked well—early involvement, diversity of profiles, and iterative documentation—alongside needs of the CAG itself: clearer and earlier articulation of CAG roles and decision latitude; a concise onboarding package (plain-language summary, glossary, timelines); predictable scheduling with adequate pre-read time and upfront clarity on recognition/expenses (set to “none” in this project). Overall, in a complex multicenter inpatient context, PPI delivered practical, low-cost modifications intended to improve the clarity and safety of the materials and a replicable pathway for similar hospital projects.

### 3.4. Contribution and Outputs

**Figure 1:**
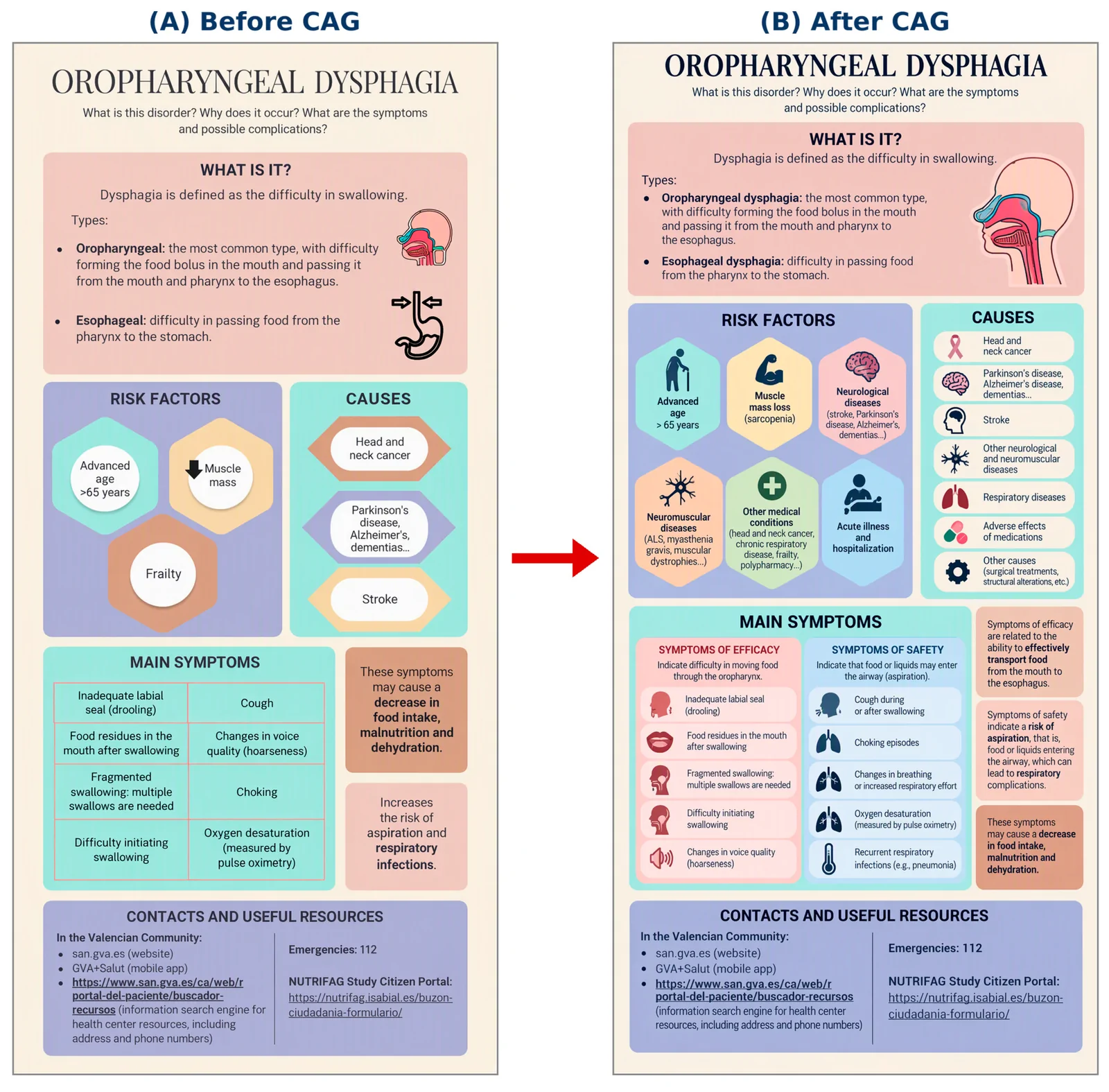
Example of a NUTRIFAG dissemination infographic on oropharyngeal dysphagia before (**A**) and after (**B**) the Citizen Advisory Group’s review. Following the contributors’ comments, the symptoms were reorganized into ‘symptoms of efficacy’ and ‘symptoms of safety’, the risk factors and causes were expanded and paired with explanatory icons, an anatomical illustration was added, and the signposting to information resources was clarified.

The process yielded tangible outputs and refinements. Dissemination resources were strengthened through clearer and more accessible messaging, the addition of a reading-easy layer, more prominent posture cues, a cleaner separation of signs from symptoms, the incorporation of QR codes linking to information sources, the removal of brand images, and video enhancements including full-screen display and captions. An ambiguity regarding communication of the project’s purpose was noted as a challenge to be addressed. A representative example of these PPI-driven changes in a dissemination infographic is shown in Figure 1.

These outputs can be considered at multiple levels. For the research team and process, early PPI engagement enabled the team to identify potential usability gaps before materials were finalized and prioritized practical cues. For intended users, the modifications were intended to make the materials more comprehensible and easier to act on for older inpatients and caregivers.

**Table 1:** GRIPP2-LF reporting checklist for patient and public involvement in the NUTRIFAG case study.

Section & Topic	Item	NUTRIFAG PPI
**1. Abstract of paper**	1a. Aim	To describe how a CAG was designed and integrated across the NUTRIFAG project phases, and to report the practical outcomes and lessons learned from its implementation in a multicenter hospital setting.
	1b. Methods	Community Case Study anchored in a Spanish multicenter hospital project on nutrition and dysphagia in adults ≥65 years; PPI evidence consolidated from CAG minutes/notes, interviews/focus groups, structured questionnaires, and e-mail exchanges; extraction mapped to GRIPP2-LF.
	1c. Results	PPI clarified design procedures and produced documented refinements to public-facing outputs (website/infographics/videos).
	1d. Conclusions	PPI was feasible and useful in a real-world multicenter hospital context, yielding documented refinements and transferable lessons for geriatric nutrition/dysphagia.
	1e. Keywords	Patient Participation; Community Participation; Deglutition Disorders; Nutritional Status; Aged.
**2. Background to paper**	2a. Definition	PPI followed the NIHR principle of research conducted with/by the public (not to/about/for); contributors included patients/caregivers, patient associations, clinicians, managers, and hospital catering leads.
	2b. Theoretical underpinnings	Interpretation used contemporary PPI literature on participation levels (information, consultation, collaboration, partnership/alliance) and roles (advisor, personal participant, co-investigator).
	2c. Concepts and theory development	Levels/roles framed degree of influence and contribution type at each phase.
**3. Aims of paper**	3a. Aim	To integrate the perspectives of patients, family caregivers, patient associations, healthcare professionals, hospital management, and catering leads into the methodological, procedural, and dissemination aspects of the NUTRIFAG project, with the aim of improving the relevance, usability, and clarity of its outputs for older hospitalized adults and their caregivers.
**4. Methods of paper**	4a. Design	PPI case embedded in NUTRIFAG, a prospective, observational, multicenter project coordinated by ISABIAL, enrolling older adults (≥65 years) in public hospitals’ medical/surgical wards.
	4b. Patient pathway / Participants	CAG composition (*N* = 17): patients/family caregivers (*n* = 3) [1 patient; 2 family/caregivers]; patient-association representatives (*n* = 3); healthcare professionals (*n* = 5) [1 speech-language pathology/phoniatrics; 1 physiotherapy; 1 nursing care assistant (TCAE); 1 social work; 1 dietetics-nutrition]; health-management/administration (*n* = 3) [care management; economic management; coordination]; hospital food & catering leads (*n* = 3).
	4c. Stages of involvement	Design and dissemination.
	4d. Level or nature of involvement	Activities spanned information (briefings), consultation (document reviews, usability feedback), collaboration (iterative co-redesign).
	4e. Capture/measurement of PPI impact	GRIPP2-LF.
	4f. Rigor of methods used	Consolidation within GRIPP2-LF; consensus between the research team and the academic researchers in charge of the CAG; no separate analytic framework beyond GRIPP2-LF.
**5. Economic assessment**	5a. Economic assessment	Not performed; no compensation/reimbursement to public contributors; virtual format minimized logistical costs.
**7. Study results**	7a. Reported results (positive/negative)	Positive: clearer messaging; reading-easy layer; posture prominence; separate signs vs. symptoms; QR codes; removal of brands; full-screen player + captions; clarified design procedure. Challenges: initial ambiguity about project purpose.
	7b. Outcomes of PPI	Finalized/updated website and infographics (A4 printable ‘do/don’t’), quick-reference thickener table (equivalences/viscosities), video player enhancements (full-screen + captions).
	7c. Impacts of PPI	On team/process: earlier detection of usability gaps; prioritization of practical aids and cues. On intended users/contributors: modifications intended to make materials more comprehensible and easier to act on for older inpatients and caregivers (not formally tested among end users).
	7d. Context of PPI	Multicenter public-hospital setting (nine hospitals, seven autonomous communities) with older inpatients (≥65 years) in medical/surgical wards; ethics in place.
	7e. Process of PPI	Three virtual meetings (~60 min each) within which semi-structured interview prompts and focus-group facilitation were conducted; iterative follow-up via e-mail for drafting and approvals.
	7f. Management of PPI	CAG operated as a standing advisory structure coordinated by the investigative team; minutes and tracked-change drafts maintained; no compensation.
	7g. Role of the public in the research cycle	Co-advisory in design and co-review in dissemination.
	7h. Re-evaluation	No formal post-implementation re-test.
	7i. Measurement	Impact was evidenced through documented changes in study and dissemination outputs and their mapping to GRIPP2-LF items. No formal post-implementation retest of comprehension or clinical effect was undertaken. Contributors’ experience of the participatory process was explored qualitatively at an in-person meeting.
**8. Discussion and conclusions**	8a. Outcomes and conclusions	In a complex inpatient context, PPI delivered practical, low-cost modifications intended to improve clarity and safety and a replicable pathway for future hospital projects.
	8b. Definition (consistency with PPI used)	PPI is consistent with NIHR (‘with/by’ the public) and was embedded across design and dissemination phases.
	8c. Theoretical underpinnings	Activities mapped to participation levels and roles recognized in contemporary PPI literature (inform/consult/collaborate/partner; advisor/personal participant/co-investigator).
	8d. Generalisability/transferability	The CAG model may be transferable to other hospital services managing nutrition/dysphagia in older adults, with local adaptation (language, accessibility, multimedia).
	8e. Ethical issues	Conducted under host-project ethics.
	8f. Economic assessment	Not assessed; virtual format and existing governance minimized added costs.
	8g. Reflections/critical perspective	Worked well: early involvement, diverse profiles, iterative documentation. Needs: clearer and earlier articulation of the CAG’s roles/decision latitude; a brief onboarding pack (plain-language summary, glossary, timelines); predictable scheduling with sufficient pre-read time.

CAG, Citizen Advisory Group; GRIPP2-LF, Guidance for Reporting Involvement of Patients and the Public, version 2, Long Form; ISABIAL, Alicante Institute for Health and Biomedical Research; NIHR, National Institute for Health and Care Research; PPI, patient and public involvement; QR, quick response; TCAE, nursing care assistant.

## 4. Discussion

The findings of this study suggest that a standing advisory structure, such as the 17-member CAG used in this project, can generate practical and low-cost improvements within a complex multicenter inpatient setting. The diversity of the group, comprising patients, caregivers, patient associations, healthcare professionals, management staff, and catering leads, reflects stakeholder compositions described in previous research. Bringing together individuals with different perspectives facilitates the identification of service gaps and supports the development of feasible operational improvements [24,25]. In this sense, the CAG functioned less as a consultative add-on and more as an embedded participatory component that helped shape how study outputs were developed.

The use of PPI during the design and dissemination phases also aligns with recommendations that emphasize the importance of engaging affected stakeholders early in the research process. Such proactive engagement can help accelerate the translation of findings into practice and ensure that outputs are meaningful to end users [26]. By moving along a continuum from information sharing to active collaboration, the process adopted in this study reflects collaborative approaches reported in other healthcare improvement initiatives, including programs where PPI contributed to the co-design of clinical tools and educational resources through iterative meetings [27].

Several of the refinements proposed by the CAG, including clearer messaging, the addition of easy-to-read layers, and improvements to video content, are consistent with current evidence on accessible health communication. Previous studies have highlighted the value of applying Universal Design for Learning principles to develop resources that accommodate different ways of accessing and processing information [25]. Similarly, work on the co-creation of patient educational materials has emphasized the importance of simplifying language, improving visual communication, and ensuring that materials remain culturally and contextually relevant for patients [24]. The integration of QR codes and the separation of specific signs and symptoms in the materials further reflects a “designing for dissemination” approach, in which research outputs are intentionally adapted to formats that facilitate real-world uptake [26].

These refinements were intended to generate benefits at several levels. For the research team, early feedback helped identify usability gaps, supporting previous findings that PPI can enhance the relevance and appropriateness of research outputs and inform the analytical process itself [28]. For end users, improving the clarity and usability of materials is particularly important given that limited health literacy continues to represent a major barrier to patient engagement and education [25]. This is especially relevant in hospital environments, where providing accessible information to patients and caregivers is essential for reducing risks and supporting quality of life [29].

From an operational perspective, the use of a virtual format and the absence of financial compensation presented both advantages and limitations. Virtual participation reduced logistical barriers and costs, reflecting recent observations that digital platforms can facilitate stakeholder engagement. However, this format may also introduce barriers related to digital literacy or access [24]. In addition, the decision not to provide financial compensation differs from much of the PPI literature, where remuneration is frequently described as an important facilitator of sustained engagement [30]. Limited resources may place additional burdens on both participants and researchers, highlighting the value of establishing transparent expectations regarding recognition and potential expenses in future initiatives [28].

Finally, lessons emerged from the reflective evaluation of the process. The need for clearer role definitions and improved onboarding materials suggests that early orientation is critical when involving diverse stakeholders in research activities. Similar recommendations have been reported in studies examining complex interventions, which stress the importance of structured guidance and well-defined processes to maintain engagement and support collaborative problem-solving [28,31]. Furthermore, the occasional ambiguity regarding the project’s objectives illustrates a common challenge in participatory research: aligning expectations and establishing a shared vision from the outset [32].

Several considerations should be taken into account when interpreting these findings. The effect of the PPI-driven modifications on comprehension, usability or safety was not formally assessed among end users, so the reported benefits should be interpreted as intended effects pending formal confirmation. The advisory group was weighted towards professional and organizational stakeholders and is best understood as a mixed, multistakeholder structure. The predominantly virtual, voluntary and unpaid format may have favored more motivated or digitally able contributors and departs from remuneration guidance such as the NIHR payment guidance [20]. Contributors’ experience of involvement was explored only qualitatively, without a validated instrument. Finally, involvement focused mainly on study procedures and dissemination materials and not on core clinical methods, and the study was confined to a single Spanish multicenter project; as our aim was in-depth description and not statistical generalization, transferability should be assessed locally. Being aware of these considerations allowed us to adopt strategies to minimize their potential impact and to safeguard the robustness of the results.

## 5. Conclusions

This study shows that embedding a diverse, multistakeholder advisory group was feasible in a multicenter hospital project. The CAG generated documented modifications to the project outputs that were intended to make them clearer, more accessible, and better aligned with the needs of patients and caregivers.

Early involvement of stakeholders during the design and dissemination phases helped detect usability issues and improve the relevance of the materials. At the same time, the experience highlighted the importance of clearly defining roles, providing adequate onboarding, and ensuring transparent expectations for participation.

Overall, structured PPI was feasible in this context and produced documented refinements to communication materials; whether these refinements objectively improve comprehension, usability, safety or clinical outcomes remains to be established in future studies with formal end-user evaluation.

## Data Availability

The qualitative documentary materials generated and analyzed during the PPI process (meeting minutes, written comments, questionnaire responses, and successive versions of the dissemination materials) are available from the corresponding author upon reasonable request; restrictions apply to preserve the confidentiality of contributors. The completed GRIPP2-LF reporting checklist supporting the findings is provided in Table 1.

## Appendix A. The NUTRIFAG Group

The members of the NUTRIFAG Working Group who contributed to the host study are the following (in alphabetical order): Ángel Luís Abad-González, Marco Aldonza-Torres, Elena Altarribas-Bolsa, Rahma Amrani, María Argente-Pla, Esther Barrufet-Alcántara, Joan Blanco-Blanco, Rafaela Camacho-Bejarano, María Ángeles Canovas Molina, Francisco Javier Carrasco-Sánchez, Cristina Carretero-Randez, Pedro Raúl Castellano-Santana, Jenifer Castellano-Santana, María Trinidad Castillo-García, Sandra Cebrián-Jimeno, Marta Charlo-Bernardos, Esperanza Ciérvide-Górriz, Rosa Ana Clement-Santamaría, María José Compañ-Aguilar, Clara De la Fuente-Gómez, Manuela Domingo-Pozo, Cristina Domínguez-Gadea, Yesmina El-Khattabi-Ofkir, Purificación Enguix-Bou, Marina Figueras-Acebillo, Ascensión Franco-Bernal, José Ángel Franco-Romero, Agnès Gabarra-Mesalles, Miguel Galeote-Muñoz, Carla García Martín, María Amparo Garrigues-Esteve, Antonia Gomariz-Martínez, María Bienvenida Gómez-Sánchez, Víctor Manuel González-Chordá, María del Pino Hernández-Plata, Beatriz Herrero-Cortina, Cristina Hurtado-Soler, Érika María Lorenzo-Ramos, Anna Marco-Mitjavila, Amando Márquez-Sixto, Idaira Martín-Santana, Laura Meseguer-Galiana, Joaquín Moncho, Mercedes del Pilar Montalván-Pelegrín, Jesica Montero-Marco, María José Morano-Torrescusa, María Julia Ocón-Bretón, María Isabel Orts-Cortés (Principal Investigator), Cristina Pérez-Bello, Laura Pérez-Navarro, Paloma Portillo-Ortega, Vanesa Ramos-Abril, Isabel Rebollo-Pérez, Beatriz Rodríguez-Ojeda, Margarita Rodríguez-Pérez, Aránzazu Ruiz-Heras-Hera, Lourdes Salinero-González, Ana Belén Sánchez-García, María Dolores Santos-Rey, Mónica Timoneda-Company, Eva María Trescastro-López, Erika Lexandra Vieira-Maroun, Fabiola Zambom-Ferraresi, Antonia Inmaculada Zomeño-Ros, and Ginesa Zomeño-Ros.
